# Low rate of initiation and short duration of breastfeeding in a maternal and infant home visiting project targeting rural, Southern, African American women

**DOI:** 10.1186/s13006-017-0108-y

**Published:** 2017-04-08

**Authors:** Jessica L. Thomson, Lisa M. Tussing-Humphreys, Melissa H. Goodman, Alicia S. Landry, Sarah E. Olender

**Affiliations:** 1 United States Department of Agriculture, Agricultural Research Service, 141 Experiment Station Road, Stoneville, MS 38776 USA; 2 Department of Medicine and Cancer Center, University of Illinois at Chicago, 416 Westside Research Office Building, 1747 West Roosevelt Road, Chicago, IL 60608 USA; 3 Department of Family and Consumer Sciences, University of Central Arkansas, 201 Donaghey, 113 McAlister Hall, Conway, AR 72035 USA

**Keywords:** Breastfeeding beliefs, Breastfeeding initiation, Breastfeeding duration, African American, Rural

## Abstract

**Background:**

Despite the benefits of breastfeeding for both infant and mother, rates in the United States remain below Healthy People 2020 breastfeeding objectives. This paper describes breastfeeding outcomes of the Delta Healthy Sprouts participants during gestational and postnatal periods. Of specific interest was whether breastfeeding intent, knowledge, and beliefs changed from the early to late gestational period. Additionally, analyses were conducted to test for associations between breastfeeding initiation and breastfeeding intent, knowledge and beliefs as well as sociodemographic characteristics and other health measures.

**Methods:**

Eighty-two pregnant women were enrolled in this project spanning three Mississippi counties. Participants were randomly assigned to one of two treatment groups. Because both groups received information about breastfeeding, breastfeeding outcomes were analyzed without regard to treatment assignment. Hence participants were classified into two groups, those that initiated breastfeeding and those that did not initiate breastfeeding. Generalized linear mixed models were used to test for significant group, time, and group by time effects on breastfeeding outcomes.

**Results:**

Breastfeeding knowledge scores increased significantly from baseline to late gestational period for both groups. Across time, breastfeeding belief scores were higher for the group that initiated breastfeeding as compared to the group that did not breastfeed. Only 39% (21 of 54) of participants initiated breastfeeding. Further, only one participant breastfed her infant for at least six months. Breastfeeding intent and beliefs as well as pre-pregnancy weight class significantly predicted breastfeeding initiation.

**Conclusions:**

Our findings indicate that increasing knowledge about and addressing barriers for breastfeeding were insufficient to empower rural, Southern, primarily African American women to initiate or continue breastfeeding their infants. Improving breastfeeding outcomes for all socioeconomic groups will require consistent, engaging, culturally relevant education that positively influences beliefs as well as social and environmental supports that make breastfeeding the more accepted, convenient, and economical choice for infant feeding.

**Trial Registration:**

clinicaltrials.gov NCT01746394. Registered 5 December 2012.

## Background

Breastfeeding for both infant and maternal health benefits is well established and advocated for by many professional and public health organizations. In particular, the American Academy of Pediatrics (AAP) recommends exclusive breastfeeding for about 6 months followed by continued breastfeeding as complimentary foods are introduced and through the first year or longer as mutually desired by mother and infant [1]. Epidemiologic evidence indicates that protective infant health effects of breastfeeding include lower risk for: respiratory tract infections; otitis media (middle ear infection); serious colds, ear and throat infections; gastrointestinal tract infections; sudden infant death syndrome; clinical asthma, atopic dermatitis, and eczema; celiac and inflammatory bowel diseases; adolescent and adult obesity; and type 1 and 2 diabetes mellitus [1]. Short term maternal health benefits of breastfeeding include decreased postpartum blood loss, more rapid involution (shrinkage) of the uterus, and reduced risk of postpartum depression [1]. Long term protective effects of breastfeeding for the mother include: reduced risk for the development of rheumatoid arthritis; lower incidence of hypertension, hyperlipidemia, cardiovascular disease, and diabetes; and decreased risk of breast and ovarian cancers [1]. As stated by the AAP, breastfeeding should be considered a public health issue and not only a lifestyle choice [1].

Despite the known benefits of breastfeeding for both the infant and mother, rates in the United States (US) remain below the Healthy People 2020 objective for the percentage of infants breastfed at six months (52% vs. the target 61%) [2]. However, other breastfeeding rates in the US are approaching the 2020 objectives – 81% for ever breastfed (vs. the target 82%), 31% for breastfed at one year (vs. the target 34%), and 22% for exclusively breastfed through six months (vs. the target 26%) [2]. Unfortunately, rates are lowest in Mississippi where only 52% of infants were ever breastfed, 24% were breastfed at six months, 11% were breastfed at one year, and 9% were exclusively breastfed through six months [2]. Maternal socioeconomic factors known to be negatively associated with breastfeeding include young age, non-Hispanic black or African American race, low income (based on poverty income ratio), receiving Special Supplemental Nutrition program for Women, Infants and Children (WIC), and less than a college education [3, 4]. Geographic disparities also are evident as women living in the southeastern US are less likely to initiate and continue breastfeeding than women in other areas of the country, and women living in rural areas are less likely to breastfeed than women in urban areas [3].

Modifiable barriers to breastfeeding include lack of knowledge, social norms, poor family and social support, embarrassment, lactation problems, and employment and childcare [4]. For example, while most women in the US are aware that breastfeeding is the best source of nutrition for almost all infants, they lack knowledge about its specific benefits [5]. Additionally, the mistaken belief that big babies are healthy babies is common among many racial and ethnic groups and may lead to supplementing breastfeeding with formula if the infant is perceived as thin [6]. Further, some mothers do not ask for help with breastfeeding due to their family’s or friends’ negative attitudes towards or contradictory information given about breastfeeding [7]. Finally, returning to work can be a significant barrier to breastfeeding for many employed mothers. The Society for Human Resource Management reported that, in 2016, only 39% of companies surveyed had an onsite lactation/mother’s room [8].

The Delta Healthy Sprouts Project was designed to test the comparative impact of two maternal, infant, and early childhood home visiting curriculums on weight status, dietary intake, and health behaviors of women and their infants residing in the rural Lower Mississippi Delta region of the US [9]. Results of the primary (gestational weight gain) and secondary outcomes in the gestational period have been reported elsewhere [10–12]. While breastfeeding was a secondary health outcome targeted for improvement, preliminary analyses indicated that differences in breastfeeding outcomes between treatment arms were not significant, likely because the benefits of breastfeeding were discussed in both curriculums. Hence, this paper describes breastfeeding outcomes of the Delta Healthy Sprouts participants during the gestational and postnatal periods without regard to treatment arm. Of specific interest was whether breastfeeding intent, knowledge, and beliefs changed from the early to the late gestational period. Additionally, exploratory analyses were conducted to test for associations between breastfeeding initiation and breastfeeding intent, knowledge and beliefs as well as sociodemographic characteristics and other health measures.

## Methods

### Design and recruitment

This was a longitudinal analysis of the Delta Healthy Sprouts participants’ breastfeeding intent, knowledge, and beliefs measured at baseline [enrollment; gestational month (GM) 4 visit] and the last gestational (GM 9) visit as well as breastfeeding behaviors (initiation and duration) in the postnatal period [postnatal month (PM) 1 through PM 12 visits]. A comprehensive description of the Delta Healthy Sprouts Project has been published elsewhere [9]. Briefly, 82 pregnant women were enrolled in this project spanning three Lower Mississippi Delta counties. Recruitment activities included publicizing the study in the local media via the distribution of flyers and brochures and active recruitment by study staff at local health clinics and medical facilities serving pregnant women and at local health fairs. Women also were referred to the study by health clinic/department staff, WIC nutritionists, social service agencies, and through word of mouth by currently enrolled participants. Inclusion criteria included: female gender; at least 18 years of age; less than 19 weeks pregnant with first, second or third child; singleton pregnancy; and resident of Washington, Bolivar, or Humphreys County in Mississippi. Participant enrollment occurred on a rolling basis; hence baseline data were collected between March 2013 and December 2014.

**Fig. 1:**
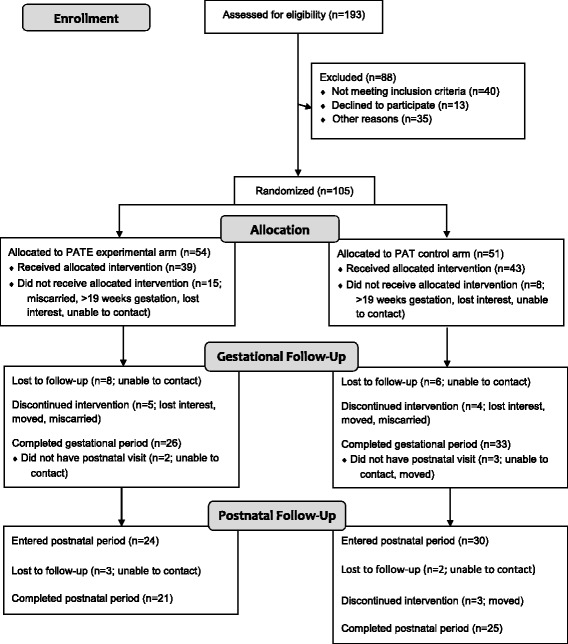
CONSORT flow diagram of recruitment, assignment, enrollment, and completion of gestational and postnatal periods

The target enrollment was 75 women in each of the two arms (control and experimental) of the project. The sample size of 150 women was based on the following assumptions: 20% attrition rate, 37% of control participants with gestational weight gain with the Institute of Medicine recommendations, and a 22% difference between treatment arms for gestational weight gain within recommendations. Additional power and sample size calculations for the postnatal primary outcomes –postpartum weight loss and child obesity at 1 year of age – were performed [9]. However, recruitment was stopped by the study’s Principal Investigator prior to reaching these numbers due to unexpected difficulties recruiting pregnant women meeting study criteria. Recruitment was extended as long as possible, but fiscal issues eventually necessitated the closing of this period. Data collection was completed in May 2016. Figure 1 illustrates the CONSORT diagram.

Delta Healthy Sprouts was designed to evaluate the impact of the Parents as Teachers® (PAT) curriculum compared with a nutrition and physical activity enhanced PAT curriculum (PATE) on maternal gestational weight gain and postpartum weight control and childhood obesity prevention. Parents as Teachers is a nationally recognized, evidence based, home visiting program that seeks to increase parental knowledge of child development, improve parenting practices, provide early detection of developmental delays, prevent child abuse, and increase school readiness [13]. Participants were randomly assigned to one of two treatment arms [PAT control (*N* = 43) or PATE experimental (*N* = 39)]. Participants were followed for 18 months, starting at approximately 4 months gestation through 12 months postnatal. At the baseline (GM 4) visit, demographic data and anthropometric measures were collected, 24-h dietary recalls were conducted, and physical activity and other questionnaires were administered.

### Intervention

The control arm of the intervention was based on the PAT curriculum that included one-on-one home visits, optional monthly group meetings, developmental screenings, and a resource network for families. Using the PAT model, Parent Educators provided parents with evidence based information and activities during home visitation. Materials were responsive to parental information requests and were tailored to the age of the child (or gestational age of the fetus).

The experimental arm of the intervention built upon the PAT curriculum by adding culturally tailored maternal weight management and early childhood obesity prevention components. The PATE curriculum was guided by the theoretical underpinnings of the social cognitive theory [14] and the transtheoretical model of behavior change [15]. Additionally, the PATE curriculum included foundational elements from the Diabetes Prevention Program and the Infant Feeding Activity and Nutrition Trial. Elements based upon the Diabetes Prevention Program principles included a flexible, culturally sensitive, individualized educational curriculum taught on a one-to-one basis [16]. Elements taken from the Infant Feeding Activity and Nutrition Trial included anticipatory guidance and parenting support principles [17]. Anticipatory guidance involves providing practical, developmentally appropriate, child health information to parents in anticipation of significant physical, emotional, and psychological milestones [18]. Parenting support emphasizes children’s psychological and behavioral goals, logical and natural consequences, mutual respect, and encouragement techniques [19].

Intervention components of the PATE arm included appropriate weight gain during pregnancy and weight management after pregnancy, nutrition and physical activity in the gestational (mother) and postnatal (mother and infant) periods, breastfeeding, appropriate introduction of solid foods, and parental modeling of healthful nutrition and physical activity behaviors. Lessons included weight gain (gestational) and loss (postnatal) charts, hands-on activities, instructional DVDs, and goal setting and barrier reduction for both diet and exercise.

Parent Educators provided both PAT and PATE participants (regardless of breastfeeding intent) with the Parents as Teachers® handouts titled “Why Breastfeed” and “Formula Feeding” as well as the monthly newsletters that featured local breastfeeding classes (location, dates, and times). Additionally, Parent Educators offered to set up a meeting with a lactation specialist for those participants who expressed interest in or ambivalence towards breastfeeding. Hence all participants, regardless of treatment arm, were encouraged to breastfeed. However, during the GM 8 visit, PATE participants watched the *Breastfeeding with Bravado* DVD (Bravado! Designs, 2008, 25 min in length) which featured discussions with mothers who have breastfed, a mother breastfeeding, and advice from experts. During the GM 9 visit, Parent Educators again discussed the benefits of breastfeeding for both the mother and her infant with PATE participants. During this visit, discussions also included other feeding options (mixed breast and formula, and formula only), infant feeding cues, and maternal postnatal nutrition.

Both arms of the intervention were delivered in the home to women beginning early in their second trimester of pregnancy by trained, community based Parent Educators. Parent Educators were African American, college educated women residing in the target communities. They were trained to deliver the nutrition and physical activity lessons and to collect data from participants, including dietary intake, by senior research staff members who were certified master trainers in the Nutrition Data System for Research (NDSR) software. Home visits occurred monthly and were approximately 60-90 min in length for the PAT lessons, and approximately 90-120 min for the PATE lessons. Both PAT and PATE participants received incentives at every visit. Gift cards were provided at the baseline and first and last postnatal visits. Other incentives included items such as diapers and baby bottles, books, and toys. Additional details regarding Parent Educator training, study methodology, and lesson plan outlines have been published elsewhere [9].

### Measures

Anthropometric measures obtained on the participants at the baseline visit included height which was measured in duplicate using a portable stadiometer (model seca 217, seca, Birmingham, UK) and weight which was measured using a digital scale (model SR241, SR Instruments, Tonawanda, NY). Both measures were performed without shoes or heavy clothing. Pre-pregnancy body weight was self-reported. Body mass index was calculated as weight (kg) divided by height (m) squared where height was averaged if the two measurements differed. Weight also was measured at each of the 17 subsequent (5 gestational and 12 postnatal) visits.

Breastfeeding intent, knowledge, and beliefs were measured at the baseline and GM 9 visits. Breastfeeding intent was captured with the survey item “When my baby is born, I intend to” with 3 exclusive responses – breastfeed only, bottle feed formula only, and combine breastfeed with formula feed. Breastfeeding knowledge (7 items) and beliefs (12 items) were assessed using true/false statements such as “breastfeeding is healthier for babies than formula feeding” and “breastfeeding is embarrassing.” These 19 items were taken from a study conducted with pregnant WIC recipients in 18 county health departments in Mississippi [20] and the national Loving Support Makes Breastfeeding Work campaign, a mail survey of low income postpartum women that was conducted in 2000 in Mississippi [21]. For each item, one point was given if the response was in the desired way (i.e., reflected the current state of knowledge about breastfeeding) and 0 points if the response was otherwise. While these items were taken from validated instruments, internal consistency or reliability of the two scales, knowledge and beliefs, was assessed in this study. Breastfeeding initiation was assessed at PM 1 visit by recording the participant’s response to the question “Are you currently breastfeeding?” If the response was “yes” or “no – stopped” then breastfeeding was considered as initiated. If the response was “no – never started” then breastfeeding was considered as non-initiated. Breastfeeding duration was assessed at PM 2 through PM 12 visits using the same question. Duration was measured conservatively as the last month at which the participant indicated she was currently breastfeeding.

Participants also provided information regarding demographic characteristics (e.g., age, marital status, household size, education, employment, household income, insurance, prenatal care), WIC participation, health history, current health conditions, and psychosocial constructs of diet and physical activity (expectations, social support, self-efficacy, and barriers) [22–25]. Details regarding other measures and questionnaire data that were collected, but are not relevant to the present paper, have been published elsewhere [9]. All measures and questionnaires were collected or administered by trained research staff (Parent Educators) using laptop computers loaded with relevant software (i.e., Snap Surveys).

### Statistical analyses

Because there were two distinct periods in this study with associated measures of interest, analyses were run using a gestational cohort (all participants enrolled in the study; *n* = 82) and a postnatal cohort (participants who completed the gestational period and had at least one visit in the postnatal period; *n* = 54). Five participants who completed the gestational period but dropped out of the study prior to the PM 1 visit were excluded from the postnatal cohort. Further, preliminary analyses indicated that differences in breastfeeding outcomes between the two treatment arms were not significant. Hence, participants were classified into two groups, those that initiated breastfeeding (BF) and those that did not initiate breastfeeding (NBF) to determine if and what differences existed between these two groups.

Statistical analyses were performed using SAS® software, version 9.4 (SAS Institute Inc., Cary, NC). Descriptive statistics, including means, standard deviations, frequencies, and percentages, were used to summarize participants’ demographic characteristics, anthropometric measures, and breastfeeding outcomes – intent, knowledge, beliefs, initiation, and duration.

Chi square tests of association or Fisher’s exact tests (categorical measures) and two sample t tests (continuous measures) were used to assess differences between BF and NBF participants’ baseline characteristics, between gestational period study completers’ and non-completers’ baseline characteristics, and between postnatal period study completers’ and non-completers’ baseline characteristics. Gestational period study completers were defined as participants who had their GM 9 visit or those who had at least two visits in the gestational period and their PM 1 visit. The second definition was used because a substantial proportion of PAT and PATE participants (36 and 42%) who had their PM 1 visit, missed their GM 9 visit due to the early birth of their infant. Postnatal period study completers were defined as participants who had their PM 12 visit.

To determine the structure or relationships within the sets of breastfeeding knowledge and beliefs items, multiple correspondence analysis was used. Correspondence analysis is conceptually similar to principal component analysis, but applicable to categorical rather than continuous data. It provides a means of graphically representing the structure of cross tabulations to help in elucidating underlying mechanisms [26]. Item profiles that fall in approximately the same direction away from the origin and are located in approximately the same region of space are associated with each other. Further, the overall spatial variation (inertia) in each set of data points is quantified and assists in the interpretation of the plot.

Generalized linear mixed models, using maximum likelihood estimation, were used to test for significant group, time, and group by time (interaction) effects on breastfeeding outcomes. Maximum likelihood estimation is an approach for handling missing data in repeated measures. Group (BF vs. NBF) was modeled as a fixed effect for all outcomes. Breastfeeding knowledge and belief outcomes were modeled using a Gaussian distribution with an identity link function and time (GM 4 and GM 9 visits) was modeled as a repeated measure using a variance covariance structure. Least squares means with 95% confidence limits were computed using these models. Due to small cell sizes, the original three categories of breastfeeding intent – exclusive breastfeeding, mixed breast and formula feeding, and formula feeding only – were collapsed into two categories – exclusive breastfeeding plus mixed breast and formula feeding vs. formula feeding only. Hence breastfeeding intent was modeled as a binomial distribution with a logit link function and time (GM 4 and GM 9 visits) was modeled as a repeated measure using an exchangeable covariance matrix structure. A sensitivity analysis that excluded the 19 (8 BF and 11 NBF) participants who completed the breastfeeding questionnaire during the PM 1 visit due to a missed GM 9 visit also was conducted. This was due to the concern that these participants may have answered the breastfeeding intent question according to their postnatal infant feeding behavior and not their intent in the last month of pregnancy.

Exploratory univariate analyses (Fisher’s exact tests and two sample t tests) indicated that relationships existed between breastfeeding initiation and breastfeeding intent at GM 4 and GM 9 and beliefs at GM 9. Hence, logistic regression was used to determine if breastfeeding initiation was predicted by breastfeeding intent (exclusive breastfeeding/mixed feeding vs. formula feeding only) and beliefs while controlling for baseline characteristics that differed between breastfeeding groups. Odds ratios with 95% Wald confidence limits (CL) were computed. The significance level of the tests was set at 0.05.

## Results

Retention rates were 72% (59/82) in the gestational period and 56% (46/82) in the postnatal period. The mean number of gestational visits was five (standard deviation [SD] = 1.3 visits) while the mean number of postnatal visits was ten (SD = 3.2 visits). Participants who missed their GM 9 visit completed the breastfeeding knowledge, beliefs, and intent survey during their PM 1 visit.

Table 1 presents descriptive statistics of baseline characteristics for the gestational cohort and comparisons between breastfeeding groups for the postnatal cohort. The majority of participants were African American (96%), single (93%), received WIC (84%), and overweight/obese prior to pregnancy (67%). Additionally, participants were young (mean age = 23 years) and early in their second trimester of pregnancy (mean gestational age = 18 weeks). Characteristics were similar between the two breastfeeding groups with the exceptions of parity, pre-pregnancy weight class, and age. Significantly more BF participants were pregnant with their first child (79%) as compared to NBF participants (39%), while significantly more NBF participants were overweight or obese before becoming pregnant (79%) as compared to BF participants (43%). Also, NBF participants were approximately 2.5 years older than their BF counterparts.

**Table 1:** Participant baseline demographic, anthropometric, and psychosocial characteristics overall and by breastfeeding initiation

	Overall		NBF (*N* = 33)		BF (*N* = 21)		
Characteristic	*n*	%	*n*	%	*n*	%	*P*
Race							1.000
African American	79	96.3	32	97.0	20	95.2	
White	3	3.7	1	3.0	1	4.8	
Marital status							0.767
Single^a^	76	92.7	29	87.9	19	90.5	
Married	6	7.3	4	12.1	2	9.5	
Education level^b^							0.349
9th-11th grade	14	17.1	7	21.2	1	4.8	
High school graduate	27	32.9	6	18.2	10	47.6	
Some college/technical	30	36.6	14	42.4	6	28.6	
College degree	11	13.4	6	18.2	4	19.0	
Employment status							0.417
Full time/part-time	29	35.4	15	45.5	6	28.6	
Unemployed (looking)	35	42.7	11	33.3	8	38.1	
Homemaker/student	18	22.0	7	21.2	7	33.3	
Household income^c^							0.555
< $500	15	18.3	6	18.2	2	9.5	
$500 -- $1,000	19	23.2	8	24.2	8	38.1	
$1,001 -- $1,500	16	19.5	9	27.3	3	14.3	
$1,501 -- $2,000	12	14.6	4	12.1	4	19.0	
$2,001 -- $4,000	9	11.0	4	12.1	1	4.8	
Don't know/refused to answer	11	13.4	2	6.1	3	14.3	
Parity^d^							0.006
First child	44	58.7	13	39.4	15	78.9	
Second child	15	20.0	8	24.2	2	10.5	
Third child	16	21.3	12	36.4	2	10.5	
Smoker in household	24	29.3	12	36.4	4	19.0	0.174
Smoker^e^							1.000
Current	4	4.9	1	3.0	1	4.8	
Stopped before pregnancy	1	1.2	1	3.0	0	0.0	
Stopped after became pregnant	2	2.4	1	3.0	0	0.0	
Non	75	91.5	30	90.9	20	95.2	
Receiving WIC	69	84.1	31	93.9	17	81.0	0.139
Gestational hypertension	5	6.1	0	0.0	3	14.3	0.054
Pre-pregnancy weight class^f^							0.007
Underweight (BMI < 18.5)	7	8.5	4	12.1	2	9.5	
Healthy weight (18.5 ≤ BMI < 25)	20	24.4	3	9.1	10	47.6	
Overweight (25 ≤ BMI < 30)	19	23.2	12	36.4	2	9.5	
Obese (BMI ≥ 30)	36	43.9	14	42.4	7	33.3	
	Mean	SD	Mean	SD	Mean	SD	*P*
Age (years)	23.0	4.62	24.6	5.30	22.0	3.55	0.050
Household size	3.9	1.69	4.0	1.65	3.7	1.49	0.567
Gestational age^g^	17.5	2.14	16.7	2.20	16.4	2.09	0.602

*NBF* no breastfeeding, *BF* initiated breastfeeding, *WIC* Supplemental Nutrition Assistance Program for Women, Infants and Children, *BMI* body mass index

^a^Includes 1 participant who indicated she is divorced

^b^Comparison = less than or high school graduate vs. some college or greater

^c^Comparison performed as continuous variable due to large number of classes

^d^Comparison = non vs. 1 or 2

^e^Comparison = non vs. all other responses

^f^Based on self-reported pre-pregnancy weight; comparison = underweight or healthy weight vs. overweight or obese

^g^Based on reported due date; enrollment data collected late for 3 participants

Regarding completion status for the gestational period, baseline comparisons revealed significantly more study completers owned or had access to a motor vehicle as compared to non-completers (95 vs. 78%, *p* = 0.036). This difference was no longer significant for baseline comparisons between postnatal period study completers and non-completers (96 vs. 83%, *p* = 0.130).

### Breastfeeding outcomes in the gestational period

Based on the correspondence plots, correct responses for the knowledge items appeared to cluster in the same region of the graph, while incorrect responses clustered together in another region. Likewise, generally more positive belief responses appeared to cluster in the same region of the graph, while more negative belief responses clustered in another region. The total inertia (variability) accounted for by the first two principal axes was 99.9% for knowledge items and 76.7% for beliefs, indicating that the two-dimensional approximation provided a good representation of the data points. Based on these analyses, it was concluded that both the knowledge items and the belief items likely represented one underlying construct, respectively, in the data. Hence knowledge and belief scales were created by summing the responses in each set of items.

At baseline, 98% (80 out of 82) of participants indicated that breastfeeding is healthier for babies than formula feeding. Correct responses for the other six knowledge items ranged from 33% (breastfeeding will make it less likely that I will get breast cancer) to 76% (breastfeeding will help me to lose weight after pregnancy). Breastfeeding knowledge significantly increased from baseline to GM 9 (4.6 to 5.6 out of 7 points, *p* < 0.001). Also at baseline, 95% (78 out of 82) of participants indicated that they did not believe that breastfeeding would make people think they did not have any money. Positive responses for the other 11 belief items ranged from 33% (breastfeeding hurts; false response) to 93% (breastfeeding is nasty; false response). The increase in breastfeeding beliefs from baseline to GM 9 was not significant (8.3 to 8.6 out of 12 points, *p* = 0.291). Likewise, participants were equally as likely to indicate their intent was to exclusively breastfeed or mixed breast and formula feed their infant vs. formula feed only at baseline (47 vs. 53%) as compared to GM 9 (55 vs. 45%, *p* = 0.900).

Table 2 presents the results for the breastfeeding knowledge and beliefs analyses by breastfeeding group and gestational visit. Breastfeeding knowledge mean scores for NBF participants at GM 4 and GM 9 were 4.4 and 5.6 out of 7 points, respectively; corresponding scores for BF participants were 4.9 and 5.6 points, respectively. These scores reflect relatively high knowledge scores for both groups of participants at both time points. Time was a significant effect across groups with higher scores observed at GM 9 as compared to baseline. Breastfeeding beliefs mean scores for NBF participants at GM 4 and GM 9 were 7.8 and 7.9 out of 12 points, respectively; corresponding scores for BF participants were 8.6 and 9.5 points, respectively, reflecting a moderately positive belief level for both groups of participants at both time points. A significant group effect was found indicating that BF participants held more positive beliefs about breastfeeding as compared to NBF participants at both time points.

**Table 2:** Breastfeeding knowledge and beliefs in the gestational period by group and visit (time)

	No Breastfeeding				Initiated Breastfeeding				*P*		
Visit	n	LSM	95% CL		n	LSM	95% CL		Time	Group	Int
*Knowledge (range 0-7)*											
GM 4	31	4.4	3.9	5.0	20	4.9	4.2	5.6	0.005	0.525	0.403
GM 9	30	5.6	5.1	6.2	19	5.6	4.8	6.3			
*Beliefs (range 0-12)*											
GM 4	31	7.8	7.2	8.5	20	8.6	7.8	9.4	0.178	0.002	0.297
GM 9	30	7.9	7.3	8.6	19	9.5	8.7	10.4			

*LSM* least squares mean, *CL* confidence limit, *Int* interaction, *GM* gestational month

Table 3 presents the results for the breastfeeding intent analyses by breastfeeding group and gestational visit. Six of 8 (75%) NBF participants who indicated at baseline that their intent was to exclusively breastfeed or mix breast with formula feed their infant indicated the opposite (formula feed) at GM 9. Conversely, only 2 of 14 (14%) BF participants who indicated at baseline that their intent was to exclusively breastfeed or mix breast with formula feed their infant indicated the opposite at GM 9. Considering the opposite change, 9 of 20 (45%) NBF participants who indicated at baseline that their intent was to formula feed their infant indicated exclusive breastfeed or mix breast with formula feed at GM 9. Similarly, 3 of 5 (60%) BF participants who indicated at baseline that their intent was to formula feed their infant indicated exclusive breastfeed or mix breast with formula feed at GM 9. Across time, significantly more BF participants indicated their intent was to exclusive breastfeed or mix breast with formula feed their infant as compared to NBF participants. Percentages did not change substantively for the sensitivity analysis although the group effect lost significance (*p* = 0.054) likely due to the reduction in sample size. Further, the proportions of participants whose postnatal infant feeding behavior matched their reported infant feeding intent at GM 9 (or PM 1) was not significantly different between participants who did and did not have their GM 9 visit (63 vs. 74%, *p* = 0.452).

**Table 3:** Breastfeeding intent in the gestational period by group and visit (time)

	GM 9 Intent^a,b^										
	No Breastfeeding				Initiated Breastfeeding						
	Exclusive + Mix		Formula		Exclusive + Mix		Formula		*P*		
GM 4 Intent^a^	n	%	n	%	n	%	n	%	Time	Group	Int
Exclusive + Mix	2	7.1	6	21.4	12	63.2	2	10.5	0.460	0.011	0.988
Formula	9	32.1	11	39.3	3	15.8	2	10.5			
	GM 9 Intent^a,c^										
	No Breastfeeding				Initiated Breastfeeding						
	Exclusive + Mix		Formula		Exclusive + Mix		Formula		*P*		
GM 4 Intent^a^	n	%	n	%	n	%	n	%	Time	Group	Int
Exclusive + Mix	1	5.9	1	5.9	8	72.7	0	0.0	0.146	0.054	0.501
Formula	8	47.1	7	41.2	2	18.2	1	9.1			

*GM* gestational month, *Int* interaction

^a^Original 3 categories -- exclusive breastfeeding, mixed breast and formula feeding, and formula feeding only -- collapsed into 2 categories with exclusive and mixed combined

^b^Included all participants who completed the gestational period

^c^Excluded 19 participants who missed their GM 9 visit and thus completed the breastfeeding questionnaire during their postnatal month 1 visit

### Breastfeeding outcomes in the postnatal period

Of the 54 participants, 21 (39%) indicated they had initiated breastfeeding, although only 10 (48%) of these 21 participants were currently breastfeeding at the PM 1 visit. Only 2 participants continued breastfeeding their infants until at least PM 3 and only 1 participant exclusively breastfed her infant and did so for 7 months. All other participants who breastfed also fed their infants formula.

Breastfeeding intent at GM 4, beliefs at GM 9, and pre-pregnancy weight class significantly predicted breastfeeding initiation (84% concordant). Participants who indicated their intent was to exclusively breastfeed or mix breast with formula feed their infant at GM 4 were 6.0 times (95% Wald CL = 1.38 to 27.25) as likely to initiate breastfeeding than participants who indicated their intent was to formula feed their infant at GM 4. Additionally, for every 1 point increase in the mean breastfeeding beliefs score at GM 9 (mean score = 8.5), the odds of initiating breastfeeding increased by 1.6 (95% Wald CL = 1.02 to 2.42). Further, participants classified as underweight or at a healthy weight prior to becoming pregnant were 5.6 times (95% Wald CL = 1.14 to 27.12) as likely to initiate breastfeeding as compared to their overweight or obese counterparts. Participant age and parity were not significant model covariates for breastfeeding initiation.

## Discussion

This paper is unique in that it reports breastfeeding initiation rates for a population of women with several predisposing characteristics for non-initiation of breastfeeding. Previous studies have reported initiation rates of 52% for residents of Mississippi, 61% for African American women overall, and 46%, 41%, 27% for African American women receiving WIC, not married, and living in rural communities, respectively [2, 27, 28]. The breastfeeding initiation rate of 39% observed for Delta Healthy Sprouts participants is lower than all these rates, in some instances substantially, with the exception of the rate for rural African American women. Additionally, only one participant breastfed her infant for at least six months. These numbers remain well below the Healthy People 2020 objectives of 82% for ever breastfed and 61% for breastfed at six months [2]. Clearly, breastfeeding remains a significant public health concern in this population of rural, Southern, predominantly African American, and low income women.

The significant increase in breastfeeding knowledge was encouraging given that the PAT curriculum advocates for and includes information about breastfeeding. Conversely, the lack of significant increases in breastfeeding beliefs was unexpected and indicates that the information and materials provided to participants were not sufficient to positively affect such beliefs. Additionally, the low number of participants who changed their mind (stated intent at GM 4) and initiated breastfeeding with their infant (12%) suggests that increasing knowledge about the benefits of breastfeeding may not be sufficient to positively affect behavior. Changing beliefs also is likely necessary to enable women to try breastfeeding their infant. This supposition is supported by the positive association between breastfeeding beliefs and odds of initiating breastfeeding observed in the current study.

Other researchers also have reported positive associations between beliefs aligned with current breastfeeding recommendations and initiation of breastfeeding. In a telephone survey study conducted with 733 low income women in Mississippi, positive attitudes or beliefs about the benefits of breastfeeding for both mother and infant as well as the enjoyable nature of breastfeeding for the mother were associated with increased odds of breastfeeding initiation [21]. Similarly, a cross-sectional survey of 283 WIC mothers found significant differences in beliefs towards breastfeeding between women who ever breastfed and women who never breastfed. Specifically, women who ever breastfed were more likely to agree that breastfeeding assists with losing baby weight, babies fed breastmilk are less likely to get sick, and breastfeeding helps mother bond with their babies more quickly than formula feeding as compared to women who never breastfed [29]. Positive attitudes and beliefs about breastfeeding are undoubtedly necessary for increasing a woman’s motivation and thereby influencing her intent to breastfeed her infant.

The fact that only one participant exclusively breastfed her infant is especially discouraging given the many avenues used to mitigate the known modifiable barriers to breastfeeding. Changing social norms was addressed through discussions with the Parent Educators regarding ambivalence towards breastfeeding as well as the *Breastfeeding with Bravado* DVD (i.e., breastfeeding in public, particularly with an older infant, and breastfeeding at work), albeit the DVD was shown only to PATE participants. Social support was targeted through the provision of information regarding local breastfeeding classes as well as referrals to lactation specialists. A more in-depth review of the individual breastfeeding beliefs items suggests that male partners and female family members of these women should have been targeted for support. Significantly more participants who breastfed indicated that their baby’s father wanted them to breastfeed as compared to participants who did not breastfeed (42 vs. 10%, *p* = 0.013). Conversely, significantly less participants who breastfed indicated that other (than their mother) women in their family thought they should only feed their infant formula (5 vs. 35%, *p* = 0.018). It also is probable that these efforts were not successful in part due to other immutable or harder to change risk factors for non-breastfeeding that were prevalent in this cohort of women, including pre-pregnancy obesity [30], African American race, non-married status, relatively low educational attainment, and WIC eligibility [21]. Clearly, a more concerted and comprehensive effort is needed to improve breastfeeding rates and increase breastfeeding duration in this population of women.

The longitudinal design of this study is one of its greatest strengths as it allowed for the capture of change in breastfeeding outcomes for both the gestational and postnatal periods. Additionally, the population studied is a strength given that African American women have low rates of breastfeeding initiation and six-month duration. However, several limitations bear mentioning as well, most notably the small sample size that limited the ability to detect statistically significant differences between the two breastfeeding groups. Data collection was not blinded and therefore a potential source of bias. However, because the data was collected in the participants’ homes, it was not practically, logistically, or financially feasible to have a second set of blinded research staff whose purpose was solely to collect data. Further, given the lack of effect found in this study, it is unlikely that bias occurred on the part of the Parent Educator or the participant (e.g., provision of socially desirable responses). Finally, the high attrition rate may limit generalizability of these study results. However, the rates reported in the current study are similar to those reported in previous nutrition and/or physical activity interventions with postpartum women. Retention rates ranged from 69% in a 12-week counseling intervention [31] to 38% in a 12-month facilitated discussion group with personalized feedback on self-monitoring intervention [32].

## Conclusion

Our findings indicate that increasing knowledge about and addressing barriers for breastfeeding were insufficient to empower rural, Southern, primarily African American women residing in three Lower Mississippi Delta counties to initiate or continue breastfeeding their infants. These women were encouraged to breastfeed through multiple sources including PAT program handouts, the PATE intervention (for those randomized to this treatment), and WIC nutritionists (for those receiving this service). However, the social support provided via breastfeeding classes and lactation specialists would not have a beneficial effect if these services were not used. Further, the parent educators in this intervention either did not have children or were mothers who chose not to breastfeed their children. Hence, future interventions of this type should consider the use of peer counselors with breastfeeding experience (e.g., through home visits or WIC clinics) and group prenatal education (including targeting the male partners of pregnant women) as these supports have been shown to improve breastfeeding initiation in minority women [33]. Attaining the Healthy People 2020 breastfeeding objectives for all socioeconomic groups will require consistent, engaging, culturally relevant education that positively influences beliefs as well as social and environmental supports that make breastfeeding the more accepted, convenient, and economical choice for infant feeding.

## Abbreviations

AAP: American Academy of Pediatrics
CL: Confidence limit
GM: Gestational month
NDSR: Nutrition Data System for Research
PAT: Parents as Teachers
PATE: Parents as Teachers Enhanced
PM: Postnatal month
SD: Standard deviation
US: United States
USDA: United States Department of Agriculture
WIC: Special Supplemental Nutrition program for Women, Infants and Children
